# Study on exogenous application of thidiazuron on seed size of *Brassica napus* L.

**DOI:** 10.3389/fpls.2022.998698

**Published:** 2022-09-06

**Authors:** Lu Zhai, Lingli Xie, JinSong Xu, Benbo Xu, Jing Dong, XueKun Zhang

**Affiliations:** ^1^Engineering Research Center of Ecology and Agricultural Use of Wetland, Ministry of Education, Hubei Key Laboratory of Waterlogging Disaster and Agricultural Use of Wetland, Yangtze University, Hubei, China; ^2^College of Life Science, Yangtze University, Hubei, China; ^3^College of Agriculture, Yangtze University, Hubei, China; ^4^Hubei Key Laboratory of Food Crop Germplasm and Genetic Improvement, Hubei, China

**Keywords:** *Brassica napus* L., thidiazuron, seed size, yield, gene expression

## Abstract

Thidiazuron (TDZ) is a novel and efficient cytokinin commonly used in tissue culture, and numerous studies have demonstrated that TDZ can increase berry size. However, no study to date has explored the effect of TDZ on seed size of *Brassica napus* and the mechanism. To shed light on the effect of TDZ on the seed size of *B. napus*, four different concentrations of TDZ were applied to *B. napus*. Results indicated that TDZ treatment could increase the seed diameter and silique length of *B. napus* to varying degrees and 100 and 200 μmol/L TDZ treatments were the most effective with a 3.6 and 4.6% increase in seed diameter, respectively. In addition, the yield of *B. napus* was also substantially increased under TDZ treatment. On the other hand, confocal micrographs of embryos and cotyledon cells suggested that embryos and their cotyledon epidermal cells treated with 200 μmol/L TDZ were obviously larger in size than the control. Furthermore, TDZ promoted the upregulation of some key maternal tissue growth-related genes, including two G-protein signaling genes (*AGG3* and *RGA1*) and two transcriptional regulators (*ANT* and *GS2*). The expression analysis of genes related to the auxin metabolic pathways, G-protein signaling, endosperm growth and transcriptional regulators confirmed that treatment with TDZ negatively regulated the key genes *ABI5*, *AGB1*, *AP2*, *ARF2*, and *ARF18* during bud development stage and florescence. The results strongly suggested that TDZ might regulate the transcriptional levels of key genes involved in auxin metabolic pathways, G-protein signaling, endosperm growth and transcriptional regulators, which resulted in bigger cotyledon epidermal cells and seed size in *B. napus*. This study explored the mechanism of TDZ treatment on the seed size of *B. napus* and provided an important reference for improving rapeseed yield.

## Introduction

*Brassica napus* (AACC, 2*n* = 38) is not only an important oilseed but also a valuable biofuel. It is one of the main sources of natural edible vegetable oils in people’s daily life and has gradually become the green energy that people urgently need now. Nevertheless, owning to the dramatically increase of world population and sharply decrease in arable land area, although rapeseed has been widely cultivated all over the world, the supply of oilseed crop still can’t adequate to the production demand.

Three direct determinants of oilseed rape yield include seed number per silique, silique number per plant and seed weight. Whether rapeseed productivity can be improved or not is greatly influenced by these components (Chen et al., 2007). In addition, both seed size and seed weight were positively correlated with oilseed rape yield and the former determined the latter (Orsi and Tanksley, 2009). Furthermore, seed size and seed number are also key factors closely related to crop domestication and evolutionary fitness in a variety of plants (Butruille et al., 1999; Shi et al., 2009). When analyzing the effect of seed size on the growth and development of cotton seedlings, it was found that the growth indexes of plants cultivated with large and full seeds were better than those of small and shriveled seeds (Huang et al., 2022). Although various traits in regard to seed size are essential for yield improvement in *B. napus*, there are still few commercially available chemical reagents that can promote seed size, and the underlying mechanisms by which it regulates seed size remain unclear.

Thidiazuron (TDZ) is a highly effective synthetic phenyl urea plant growth regulator, which not only has auxin-like activity, but also a cytokinin analogue (Chang et al., 2018). Nowadays, many scholars are devoted to the application of TDZ in cotton, and its main function in cotton is to promote the shedding of cotton leaves. Recent studies found that TDZ could induce cotton leaf abscission by increasing ethylene content and the activity of cell wall degrading enzymes (Du et al., 2014). Moreover, treatments with TDZ are capable of stimulating fruit growth, accelerating fruit ripening and promoting fruit enlargement, which will greatly improve fruit quality (Famiani et al., 1999). In addition to stimulating the enlargement of grape barriers and preventing fruit drop, TDZ also had an influence on the production of aroma volatiles in viticulture (Wang et al., 2020). Treatment with 20 to 60 mg/L TDZ revealed a significant reduction in fruit drop and an increase in fruitlet retention in “Hosui” and “Packham’s Triumph” pears, especially at 60 mg/L TDZ, which resulted in the highest fruit number per tree and yields compared to other treatments (Carra et al., 2017). The same author also showed that fruit diameter and fruit weight was markedly increase in response to TDZ (Carra et al., 2021). Both of these increases should be attributed to the increasing TDZ rate, while the crop load and yield decreased linearly. Similarly, it was observed that both GA_3_ and TDZ treatments promoted fruit maturity and significantly increased berry cracking rate compared to the control treatment (Lee et al., 2013). TDZ has been widely used in agricultural production and there have been tremendous physiological and biochemical researches on it, but few studies have been conducted on the role of exogenous TDZ reatments on seed size and related pathways and mechanisms in rapeseed.

Seed size is a major trait affecting crop yield and evolutionary fitness, and it is regulated by multiple genes in different metabolic pathways and signal transduction pathways. Several recent studies have testified that seed size is regulated by the relevant signaling pathways that control maternal tissue size (Li et al., 2019). Through delving deeply into the molecular mechanisms underlying these metabolic and signaling pathways revealed that the signaling pathways controlling seed size mainly include G-protein signaling, the ubiquitin-proteasome pathway, mitogen-activated protein kinase (MAPK) signaling, HAIKU (IKU) pathway, phytohormone and some transcriptional regulators (Li and Li, 2016; Li et al., 2019). Heterotrimeric GTP-binding proteins are conserved key regulators of signaling during eukaryotes growth and development. The structural G protein is a core protein complex composed of one Gα, one Gβ and the Gγ subunits (Johnston et al., 2007; Urano and Jones, 2014; Stateczny et al., 2016). The AGG3-like-γ-subunits has a function in regulating the size of seeds and organs. AGG3-deficient mutant in *Arabidopsis* was discovered to produce smaller seeds and organs. In contrast, *AGG3* overexpression resulted in increased seed size, seed weight and seed number per plant (Li et al., 2012; Roy Choudhury et al., 2014). The typical α subunit (*RGA1*) in the heterotrimeric G protein complex has been found to be involved in the regulation of grain weight and cell function during plant development by coupling extracellular signals sensed by receptors (Biswal et al., 2022).

Plant hormones have recently been shown to play an important role in seed size regulation. In particular, the biosynthesis and signal transduction of auxin in plants controlled the seed size to a great extent (Ishimaru et al., 2013; Liu L. C. et al., 2015). The *Auxin/INDOLE-3-ACETIC ACID* (*Aux/IAA*) transcriptional repressors and the *AUXIN RESPONSE FACTOR* (*ARF*) are jointly involved in the auxin signaling process (Weijers and Friml, 2009). The seeds produced after complete loss of *ARF2* gene function were up to 46% heavier than wild-type seed parents and affected seed size through maternal tissues due to enlarged integuments (Schruff et al., 2006). *AINTEGUMENTA* (*ANT*) is a transcription factor of the AP2-domain family members that mediates cell proliferation and growth control (Klucher et al., 1996; Krizek, 2003). Studies manifested that overexpression of *ANT* gene generated larger seeds (Mizukami and Fischer, 2000). Scholars also found that the *ANT* promoter combined with *ARF2* could directly regulate the expression of *COLD-REGULATED15A* (*COR15a*), and the mutants after knocking out cor15a displayed a smaller seed size phenotype, indicating that *COR15A* positively regulated seed size (Meng et al., 2015). Evidence from polyploid *B. napus* revealed that *ARF18* determined the seed weight and silique length through an auxin-response pathway and maternal regulation. Meanwhile, the homodimers formed by *ARF18* regulated seed size by repressing auxin-responsive genes and restraining cell expansion in the silique wall (Liu J. et al., 2015). Furthermore, *BRASSINAZOLE-RESISTANT1* (*BZR1*) is considered to be an important seed growth regulator through maternal tissues (Kim et al., 2021). Some findings indicated that specific seed developmental pathways regulated by *BR* transcription resulted in altered seed size and seed shape (Jiang et al., 2013).

Transcription factors repress or enhance the expression of various genes in the process of plant growth and developmental. In particular, it plays significant role in regulating genes related to seed size, for instance, *GRAIN SIZE ON CHROMOSOME* (*GS2*), *APETALA2* (*AP2*) and *TRANSPARENT TESTA GLABRA2* (*TTG2*) (Western et al., 2001; Johnson et al., 2002; Hu et al., 2015). There are six *AP2* members in the *APETALA2*/ethylene response element binding protein (*AP2/EREBP*) transcription factor superfamily, which are mainly expressed in floral organs, ovules and seed coat development (Jofuku et al., 1994; Jofuku et al., 2005; Ohto et al., 2005, 2009). Among them, *AfAP2-2* is an *AP2* homologous gene with two conserved *AP2* structural domains. Compared with the vegetative period, its expression level is significantly higher in vegetative organs during the reproductive period. Since AfAP2-2-over-expressing *Arabidopsis* produced smaller seeds than the wild type (WT), and seed weight was also predominantly reduced relative to the WT, the above results demonstrated that *AfAP2-2* might be negatively correlated with seed size and weight (Lei et al., 2019). In summary, *AP2* is one of the major transcription factors affecting seed size and seed weight. *GS2* is a member of the *GRF* transcription factor family, which is a transcriptional activator encoding *OsGRF4* (van der Knaap et al., 2000). MicroRNA miR396c regulated *OsGRF4* in a targeted manner *in vivo*. The *GS2* mutation disrupted the interaction between *OsmiR396* and *OsGRF4*, leading to advanced expression level of *OsGRF4*, and bringing about larger grains and higher grain yield (Duan et al., 2016; Li et al., 2016).

To study the effect of TDZ on seeds, *B. napus* was treated with different concentration TDZ in this research, and the morphology and physiology of treated plants were determined. Laser confocal microscopy was used to observe embryos and cotyledon cells. Quantitative Real-time PCR (qRT-PCR) was used to measure the transcriptional levels of genes associated with maternal tissue growth (*AGG3*, *RGA1*, *ANT*, and *GS2*) and seed size (*ABI5*, *AGB1*, *AP2*, *ARF2*, and *ARF18*). The results provided the evidence that a molecular mechanism by which TDZ regulated seed size in *Brassica napus*, which greatly benefitted to the improvement of rapeseed yield.

## Materials and methods

### Plant materials and growth conditions

The field trials were conducted during growing season of 2020–2021 in Agricultural Science and Technology Industrial Park of Yangtze University, Hubei province, China (30^°^36′N 112^°^08′E). The area belongs to the subtropical monsoon humid climate zone, with an average annual temperature of 15.9–16.6°C, annual rainfall of 2600–3100 mm, and annual sunshine hours of 1800–2000 h. Changyou No. 1 cultivar of *B. napus* was used material in this study, which was a hybrid between zhongshuang 11 and a material from Czechia. It is a winter-hardy variety with high yield which is extremely sensitive to light and temperature. Completely randomized block method was used with 3 replicates and the area of each block was 18 m^2^, with a planting density of 18 cm between the plant individuals and 25 cm between rows. Field management and pest and disease prevention were conducted based on local standard practices.

The plant growth regulator TDZ were sprayed at concentrations of 10, 100, 200, and 400 μmol/L on the leaves at the early development stage in March, 3–5 days before flowering. Plants treated with distilled water were used as control. All applications of TDZ were sprayed with hand sprayer. The buds and flowers selected for sampling at the flowering stage in April 9 were picked and stored at −80°C for RT-qPCR. Siliques and seeds were harvested in May 28, 2021. At least 30 siliques and seeds from each concentration of the treatment were selected for growth and yield measurements. The harvested seeds were analyzed immediately.

### Morphological and physiological measurements of seeds

Plants were monitored from April through May 2021. It started growing siliques on April 18. Seeds ripe completely after 40 days. In May 28, 2021, final seed diameters (cm), silique length (cm) and seed number per silique were recorded. Thirty uniformly growing plants from rape treated with different concentrations of TDZ were selected for the determination of growth and yield indicators. Meanwhile all siliques of the selected plants were measured. In order to analyze the effect of TDZ on seed size, the main stem of plants was divided into three parts: top, middle and bottom, according to the branching characteristics of rape. Total number of seeds per plant was added up by using a knife to open all siliques from each plant and counting the number of seeds in it. Additionally, the number of existing seeds and empty shells number were counted. Seed setting ratio was determined as the number of existing seeds divided by the sum of the number of existing seeds and the empty shells number. Seed diameter was measured with vernier calipers. Twenty siliques were selected at the optimal concentration after TDZ treatment, and their silique lengths were measured with a ruler and photographed for comparison with the control. The seeds collected from each part of the plant were evenly mixed together and randomly selected 30 seeds from them to measure their seed diameter, then divided by the number of seeds to get the average seed diameter per plant. To calculate thousand-seed weight (g), 1,000 seeds per plant were randomly selected and weighed. Poured the seeds into the test tube to its 5 ml mark and weighed the seeds at this moment. Seed volume weight (g/ml) was determined as the seed weight divided by the volume of tick mark. The all seeds of each block was harvested separately and its yield was measured.

### Confocal laser scanning microscope observation

A dissecting needle was used to dissect CK and seeds treated with 200 μmol/L TDZ under a microscope to isolate the embryos. Seeds of 10 days after flowering and mature seeds are treated in this way. Then, the embryos placed in DIC buffer (50 mM sodium phosphate, pH 7.0/10 mM EDTA/1% Triton X-100/1% DMSO) were incubated in 37°C constant temperature incubator overnight (at least 12 h), fixed with FAA fixative (10% formalin/5% acetic acid/45% ethanol/0.01% Triton X-100). The fixed embryos were dehydrated with 30, 50, 70, 80, 90, 95, and 100% ethanol (1 h for each stage) and then treated in Hoyer’s solution (chloral hydrate: water: glycerol = 3:0.8:0.4) for 1 h (Ohto et al., 2005, 2009). The cellular morphology of the embryos was observed and photographed using a confocal laser scanning microscope observation (Leica TCS-SP8 SR, Germany) fitted with a differential interference lens (DIC). The cell number and cell size per unit area were counted by using ImageJ software (National Institute of Health, United States).

### RNA extraction and quantitative real-time PCR

RNA prep Pure Plant Plus Kit (Tiangen, Beijing, China) was used to extract the total RNA from flowers and buds under different concentrations of TDZ treatment according to the manual. In short, samples were ground with a grinder (MB-24) after freezing about 100 mg of flowers and buds in liquid nitrogen. The concentration and purity of RNA was evaluated using Ultramicro ultraviolet spectrophotometer (QuaWell Q5000, United States) by determining absorption at 260 and 280 nm. Subsequently, 1 μg RNA was reverse transcribed into cDNA using HiScript^®^ II Q RT SuperMix for qPCR Kit (Vazyme, Nanjing, China) according to the manufacturer’s protocol.

To gain insights into the response of seed size-related genes to TDZ, transcription level of nine genes involved in seed growth was detected, including an endosperm growth-related gene (*ABI5*), three transcriptional regulatory factors (*ANT*, *AP2*, and *GS2*), three G-protein signaling genes (*AGB1*, *AGG3*, and *RGA1*) and two auxin metabolic relative genes (*BnARF18* and *ARF2*). The nine genes were searched directly or BLAST in the BnPIR genome browser by using the protein sequences of *Arabidopsis* as a query. Then, Vector NTI software was used to design the specific primers for qRT-PCR accord to the genes sequences related seed development (Supplementary Table 2). Relative transcriptional level of genes associated with seed size was analyzed by qRT-PCR. The experimental protocol was carried on CFX96TM Real-Time System (Bio-Rad, United States). The total reaction solution was 10 μL, containing 5 μL 2 ×ChamQ Universal SYBR qPCR Master Mix (Vazyme, Nanjing, China), 0.4 μL forward and reverse primer, 2.6 μL autoclaved sterile water and 2 μL of 10-fold diluted template. The qRT-PCR program was conducted using three-step cycling conditions of 95°C predenaturation for 30 s, 40 cycles of 95°C for 10 s, cooling down to 60°C for 30 s and at 72°C extending for 30 s. *Actin-2* was the selection of internal control genes in rape. The relative expression levels of genes related to seed size were calculated based on the method of 2^–ΔΔ*CT*^ (Livak and Schmittgen, 2001). Each sample corresponded to three technical replicates.

### Statistical analysis

The experiment was carried out following a completely randomized devise with four replications at different concentrations of TDZ treatment. The phenotypic data of seed diameters, silique number per plant, seed number per silique, thousand-seed weight and seed volume weight were subjected to the mean ± standard error. The statistical analysis of total data was determined by applying Duncan’s Test in DPS 8.50 (Data processing software, China). A significant difference was indicated when the *p*-value < 0.05. GraphPad Prism 9.0 (GraphPad Software, Inc., United States) was used to draw figures.

## Results

### Effect of thidiazuron treatments on seed morphology of *Brassica napus*

**FIGURE 1:**
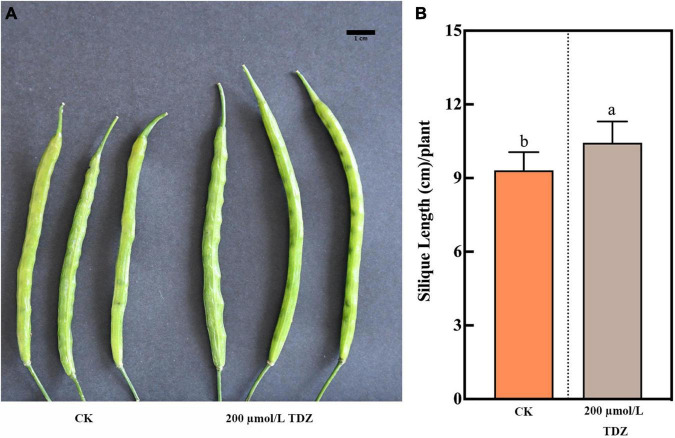
Effect of 200 μmol/L TDZ treatment on silique phenotype and silique length. **(A)** Comparisons of silique phenotype. **(B)** Comparison of silique length. Scale bar: 1 cm. Mean ± Standard error (*n* = 20). Different letters denote a significant difference (Student’s *t*-test, *p* ≤ 0.05).

**FIGURE 2:**
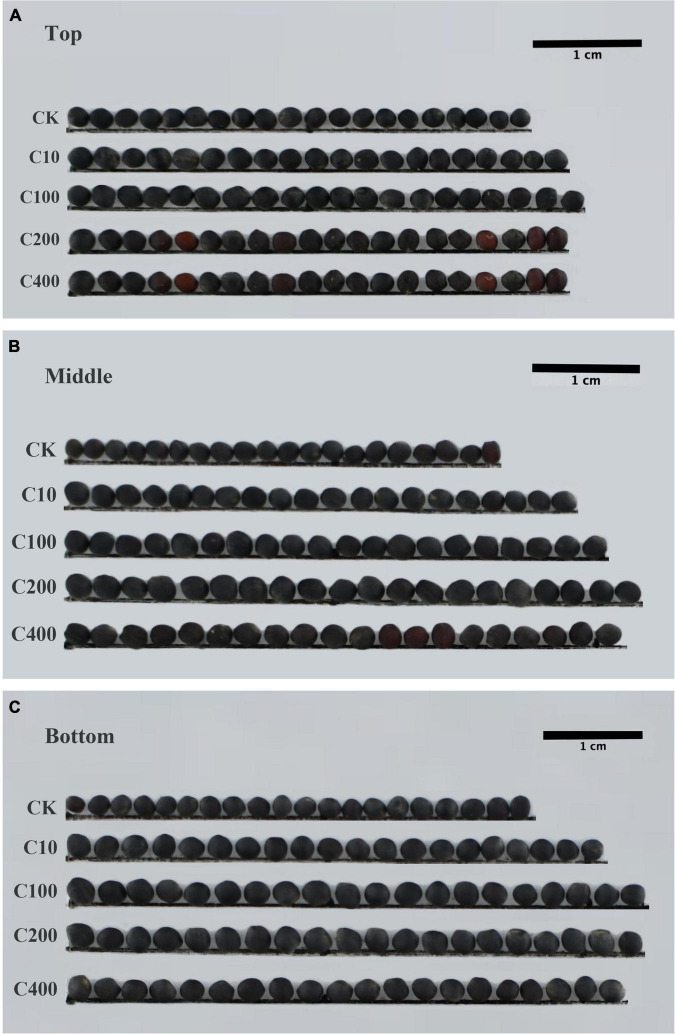
Effect of TDZ treatment on seed size of *B. napus*. **(A)** Siliques in the top; **(B)** siliques in the middle; **(C)** siliques in bottom; CK: control; C10: 10 μmol/L TDZ; C100: 100 μmol/L TDZ; C200: 200 μmol/L TDZ; C400: 400 μmol/L TDZ; Scale bars: 1 cm.

In length measurements of siliques, 200 μmol/L TDZ treatment increased the silique length located in the middle (Figure 1A). The application of 200 μmol/L TDZ achieved the greatest length (10.44 ± 0.19 cm), and control almost had a shorter siliques length, ranging from 7.70 to 10.40 cm (Figure 1B). The effect of different concentrations of TDZ on the seed size of *B. napus* at 40th day after full maturity is shown in Figure 2. It was observed that TDZ had a significant effect on seed size, as the seeds located in different parts under each concentration of TDZ treatment were obviously larger than the control. In the seeds growing in the top siliques, the seeds treated with TDZ at concentrations of 100, 200, and 400 μmol/L were greater than those of 10 μmol/L and the control (Figure 2A). The seed diameter (2.05–1.93 cm) under the all TDZ treatment was also simultaneously notably increased when compared to the control (*p* ≤ 0.05) (Supplementary Table 1). Regarding the seeds in the middle silique, all TDZ-treated seeds became larger relative to the control (Figure 2B). Meanwhile, 200 μmol/L TDZ treatment had the longest seed diameter among all TDZ-treated seeds (2.04–1.92 cm), followed by 100 and 400 μmol/L TDZ, which were different from 10 μmol/L TDZ and the control (*p* ≤ 0.05) (Supplementary Table 1). For the seeds growing in the bottom silique of the plant, the seed diameter varied from 2.05 to 1.99 cm. It was noticed that the TDZ treatment at the medium concentration (100 μmol/L) significantly increased seed size, and the seed diameter was the longest, followed by the other TDZ-treated seeds. Similarly, the seed diameter of the control remained the shortest in all cases (*p* ≤ 0.05) (Supplementary Table 1 and Figure 2C). Additionally, comprehensive analysis for seed diameter within the silique of each part of the plant showed that application of medium TDZ concentration (100 and 200 μmol/L) promoted the longer seed diameter with an increased increment of 3.6 and 4.6%, respectively relative to the control. Overall, the above-mentioned results indicated that TDZ treatment notably affected the seed size of *B. napus*.

### Effect of thidiazuron on yield-related traits in *Brassica napus*

Plants treated with TDZ showed basically consistent changes in the seed yield-related traits, including increases in thousand-seed weight, seed volume weight, the silique number per plant, yield and the seed number per silique relative to control. Thousand-seed weight is one of the important components of yield. The thousand-seed weight after treatment with 200 μmol/L TDZ was remarkably higher than control and the other concentrations of TDZ treatments. Additionally, as compared to control, the thousand-seed weight also significantly increased in the other TDZ treatment. As for seed volume weight, like thousand-seed weight, it also reached a maximum under the treatment of 200 μmol/L TDZ, while the effect of TDZ (400 μmol/L) treatment was statistically comparable to TDZ (10 and 100 μmol/L), but all of the seed volume weight treated with them was greater than that of control (Table 1).

**TABLE 1:** Effect of TDZ treatments on seed yield-related traits.

Treatment (μmol/L)	Thousand-seed weight (g)	Seed volume weight (g)	Silique number per plant	Seed number per silique	Yield (Kg/ha)
Control	2.91 ± 0.06c	0.6704 ± 0.0039b	263.25 ± 9.22b	19.01 ± 4.56c	2584.50 ± 6.56d
TDZ 10	3.27 ± 0.01bc	0.6732 ± 0.0047ab	267.25 ± 7.93b	19.77 ± 4.77bc	2666.75 ± 4.57c
TDZ 100	3.38 ± 0.04ab	0.6806 ± 0.0044ab	268.75 ± 6.24b	19.28 ± 4.70c	2763.75 ± 5.38b
TDZ 200	3.46 ± 0.04a	0.6846 ± 0.0019a	309.00 ± 6.06a	20.97 ± 4.93ab	2895.75 ± 4.57a
TDZ 400	3.16 ± 0.03d	0.6714 ± 0.0053ab	299.75 ± 4.27a	21.76 ± 3.79a	2767.00 ± 5.16b

Mean ± Standard error (SE), n ≥ 5. For each group of comparisons, thousand-seed weight, seed volume weight, silique number per plant, seed number per silique and yield values followed by the same letter are not significantly different at the 0.05 significance level based on Duncan’s multiple range test and ANOVA. The ANOVA was run for each seed yield-related traits.

Among the variables related to yield, the silique number per plant was one of the main factors, 200 and 400 μmol/L TDZ treatment significant increased silique number per plant (*p* ≤ 0.05). Moreover, the 400 μmol/L TDZ treatment outperformed the other TDZ treatments (except for the 200 μmol/L TDZ treatment) both in terms of silique number per plant and yield (*p* ≤ 0.05) (Table 1). On the other hand, the highest yield was obtained under the treatment with TDZ (200 μmol/L), followed by TDZ 100 and 400 μmol/L, TDZ 10 μmol/L also gave higher yield than the control (Table 1). The yield of the TDZ (200 μmol/L) treatment increased by 12.04% compared to the control plants. This might be attributed to the increase in seed size.

The empty shells number exhibited significance only in the siliques located in the middle, bottom and various portions of the plant. The average seed number per silique after 400 μmol/L TDZ treatment was significantly more than that in control and 100 μmol/L TDZ treatment, followed by 200 μmol/L TDZ treatment. Meanwhile, the control had the least seed number per silique in the top and middle siliques, followed by 100 μmol/L TDZ treatment. Among the siliques located in the middle, bottom and various portions of *B. napus*, the control had the most empty shells number and was significantly more than 10 μmol/L TDZ treatment. Additionally, in the siliques located in the bottom and various portion, the empty shells number in control was significantly higher than in 100 and 400 μmol/L TDZ treatments (*p* ≤ 0.05) (Supplementary Table 1). Nevertheless, the effect of TDZ treatment on the seed number per silique and the empty shells number and the relationship between the two yield traits remain to be further investigated. The seed setting ratio with low and high concentrations (10 and 400 μmol/L) TDZ treatment was notably higher in the siliques in the bottom than in the untreated *B. napus* (Supplementary Table 1). For the siliques in the bottom, the seed setting ratio after two concentrations of TDZ treatment increased by 7.39 and 7.06% compared to the control, respectively.

### Effect of thidiazuron treatments on embryo cell size and number

**FIGURE 3:**
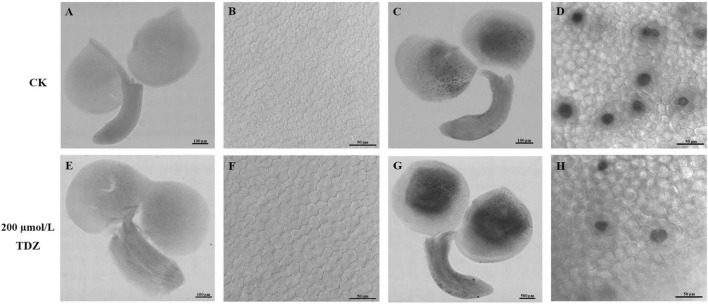
Effect of TDZ treatment on embryo cell size and number. **(A,E)** Immature embryos from seeds of 20 days after treatments; **(B,F)** epidermal cell layer from the central part of cotyledons from panels **(A,E)**; **(C,G)** mature embryos from mature dried seeds; **(D,H)** epidermal cell layer from the central region of cotyledons panels **(C,G)**. Scale bar: 100 μm in panels **(A,C,E)** and 500 μm in panel **(G)**, observation with 20× objective in DIC mode; 50 μm in panels **(B,D,F,H)**, 40× oil lens observation in DIC mode.

**FIGURE 4:**
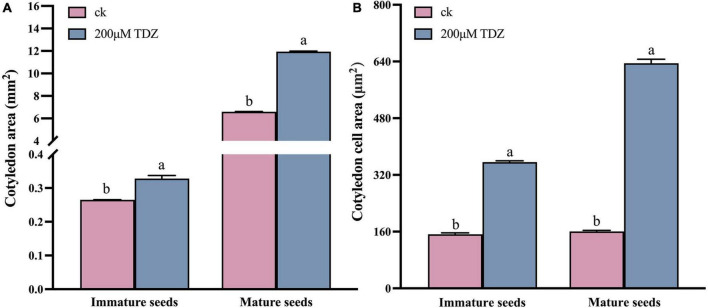
Cotyledon area **(A)** and cotyledon cell size **(B)** of immature seeds and mature seeds. Entire cotyledons from immature embryos and mature dried embryos or their central parts were cleared and photographed. Imaging analysis software was used to calculate areas. Mean ± Standard error (*n* = 3). Different values were marked with different letters at 0.05 significance level.

In order to explore the reasons for the larger seeds, a cytological analysis of the seeds was performed. The embryos were isolated and observed. The size of embryo and cotyledon cells in the siliques at 20th day after TDZ treatment is shown in Figure 3. Embryos treated with 200 μmol/L TDZ were significantly larger than that of control and the average cotyledon area in TDZ-treated embryos was 2.34 times larger than in the control (Figure 4A). Furthermore, it could be found from the laser confocal microscope image that the hypocotyl and radicle treated by TDZ was also appeared bigger than those of control (Figure 4A). Similarly, embryos in mature seeds treated with 200 μmol/L TDZ were also significantly larger than the control (Figures 3C,G), and the average area of cotyledons after TDZ treatment was 1.81 times larger than that of control (Figure 4A).

Epidermal cells in the middle of cotyledons treated with TDZ and control were selected respectively to measure the cell size. The results proved that TDZ treatments enlarged the epidermal cells of cotyledons. For the 20 days seeds after treatments, the average area of cotyledon epidermal cells was 1.24 times larger than the area of the corresponding control cells (Figures 3B,D,F,H). The cotyledon epidermal cell area in mature seeds treated with 200 μmol/L TDZ was 3.95 times larger than that of the control (Figure 4B). In immature seeds, the number of cotyledon cells treatmented with TDZ was approximately 1.89 times that of the control. Thus, TDZ treatment increased both cell size and number, which may lead to an increase in seed size as well.

### Transcriptional levels of seed size-related genes of *Brassica napus* after thidiazuron treatment

**FIGURE 5:**
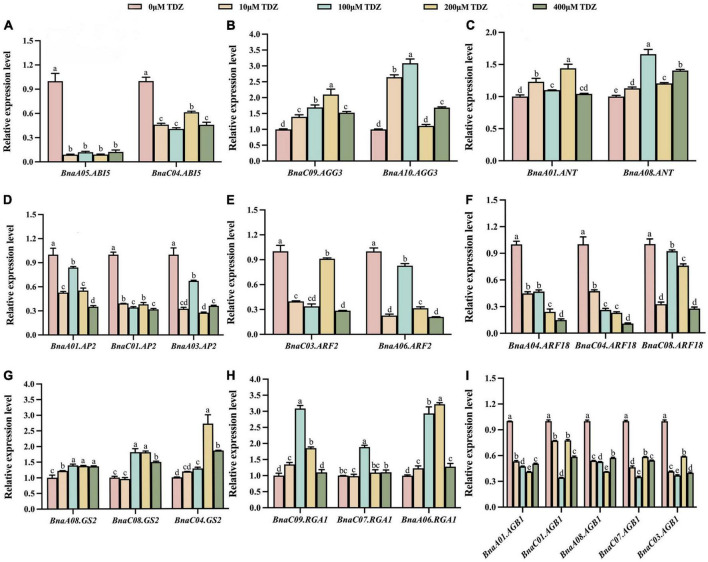
Relative transcriptional levels of seed size-related genes in buds of *B. napus*. **(A–I)** lists the relative transcriptional levels of nine genes associated with seed size located on different chromosomes in buds. Among them, the pink, orange, blue, yellow and green columns represent the relative transcriptional levels of genes in buds treated with 0 μmol/L, 10 μmol/L, 100 μmol/L, 200 μmol/L and 400 μmol/L TDZ, respectively. Three biological replicates and three technical replicates were carried out using buds from 3 to 5 days before flowering. Mean ± Standard error (*n* = 3). Different letters suggest significant differences (Duncan’s multiple range test, *p* ≤ 0.05).

**FIGURE 6:**
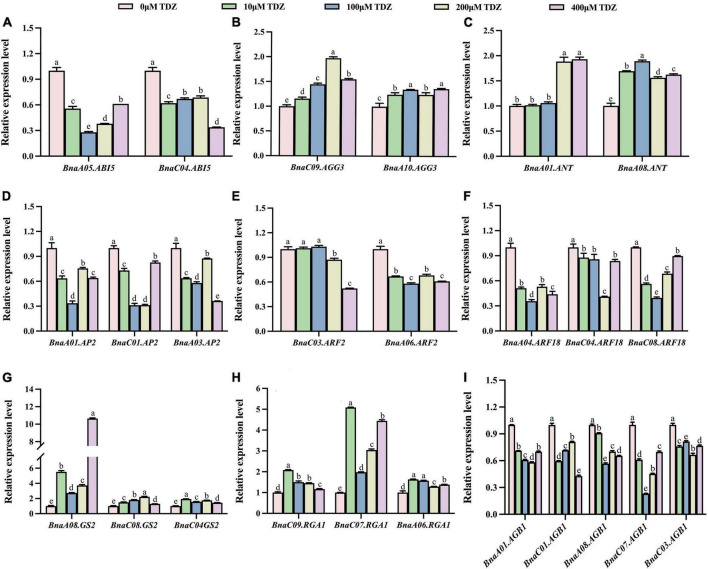
Relative transcriptional levels of seed size-related genes in flowers of *B. napus*. **(A–I)** lists the relative transcriptional levels of nine genes associated with seed size located on different chromosomes in flowers. Among them, the pink, green, blue, yellow and purple columns represent the relative transcriptional levels of genes in flowers treated with 0 μmol/L, 10 μmol/L, 100 μmol/L, 200 μmol/L and 400 μmol/L TDZ, respectively. Three biological replicates and three technical replicates were carried out using 10-day-old blooming flowers. Mean ± Standard error (*n* = 3). Different letters suggest significant differences (Duncan’s multiple range test, *p* ≤ 0.05).

As shown in Figures 5, 6, the transcriptional patterns of these genes were similar in the buds and flowers. Although the gene transcriptional levels differed slightly under different concentrations of TDZ treatment, the overall expression trends of individual genes located on different chromosomes were basically the same. The results showed that transcriptional levels of *ABI5* gene related to endosperm formation was significantly decreased in buds (Figure 5A). Compared with the control group, *AP2*, *ARF2*, *BnARF18* and *AGB1*, which played a negative regulatory role in seed size control, were also down-regulated in the same buds (Figures 5D–F,I). By contrast, compared with the control group, the transcriptional levels of the *AGG3*, *ANT*, *GS2*, and *RGA1* genes, which were positive regulators of seed size, were significantly increased after TDZ treatment in buds (Figures 5B,C,G,H). Simultaneously, the transcription of these genes showed the same trend in flowers (Figure 6). Overall, exogenous TDZ treatment induced the transcription of these genes to some extent. Interestingly, the transcriptional level of *AGG3*, *ANT*, *GS2*, and *RGA1* genes, which were all positive regulators of seed size, reached a maximum after TDZ treatment at relatively medium concentrations (100 or 200 μmol/L) in buds. In flowers, the transcriptional levels of *AGG3* and *ANT* also reached a maximum at moderate concentrations (100 or 200 μmol/L) of TDZ treatment. In contrast, compared to that of control, the transcriptional level of the genes *BnaA05.ABI5*, *AGB1* (except *BnaC01.AGB1*), *AP2* (except *BnaA03.AP2*) and *ARF18* reached a minimum when treated with TDZ at the same concentrations (100 or 200 μmol/L). Combined with the previous phenotypic and morphological results, it could be speculated that a medium concentration (100 or 200 μmol/L) of TDZ treatment may be the optimum concentration for increasing seed size. To summarize, TDZ might affect seed growth and regulate seed size by promoting or repressing the transcription of genes related to seed size.

## Discussion

### Thidiazuron can be used as a plant growth regulator to improve the yield of *Brassica napus*

Thidiazuron is considered as a substituted phenyl urea compound with strong auxin- and cytokinin-like activities. It has been proved to promote seed germination and stomatal formation on floral parts (Meng et al., 2004; Sharaf et al., 2011). What’s more, TDZ has the effect of inducing and stimulating cotyledon growth and development as well as increasing fruit weight, etc. (Guo et al., 2011). In fruit cultivation, studies have found that TDZ can effectively promote the fruit growth of various fruits such as apples (Greene, 1995), persimmons (Itai et al., 1995) and kiwis (Famiani et al., 1999; Famiani et al., 2002), increasing their size and seed setting ratio. Although many studies have demonstrated TDZ can promote fruit enlargement and increase fruit weight, there are few studies on its role in seed size.

Seed size is an important agronomic trait, which not only affects plant and environmental stress adaptation, but also crop quality and yield (Guo et al., 2020; Miao et al., 2020). Study has revealed that gibberellic acid in combination with 5 mg⋅L^–1^ TDZ increases berry width (Kimura et al., 1996). Based on previous study, TDZ treatments increased the fruit diameter led to a decrease in the fruit length/average diameter ratio, which caused a change in fruit shape, while an increase in fruit size and weight (Famiani et al., 1999). Additionally, spraying TDZ (20–30 mg⋅L^–1^) on small-fruited “Spadona” and “Coscia” pears resulted in a significant increase in fruit size (Stern et al., 2003). The fact that fruit size was increased by exogenous application of TDZ was also reported in “Hosui” pears and “Gala” apples (Petri et al., 2001; Pasa et al., 2017). Altogether, above studies indicated that TDZ had an important regulatory role on seed size. *B. napus* is one of the most important oilseed crops widely grown worldwide. It has been reported that the determinants of seed yield in oilseed crops include effective silique number per plant and seed number per silique, and thousand-seed weight depending on the seed number and size (Ozer, 2003; Jiao et al., 2012). In this study, results indicated that TDZ treatment was effective in promoting seed size in *B. napus*. 100 and 200 μmol/L TDZ treatments significantly increased seed diameter of 40-day-old siliques of *B. napus*, and other concentrations of TDZ treatment also increased seed diameter compared with the control (Supplementary Table 1). Our study proved that 200 μmol/L TDZ treatment had the most silique number per plant among all treatments (Table 1). Except for the siliques located in the bottom portion of the plant, the seed number per silique was significantly higher at a high concentration (400 μmol/L) than other concentrations of TDZ treatment, while the seed number per silique in all TDZ-treated plants was no less than in the control (Supplementary Table 1). In kiwifruit, TDZ greatly increased the fruit number with protruding distal ends at high concentrations. However, TDZ did not result in significant changes in seed number (Famiani et al., 1999). Similar results emerged from the study of small-fruited “Spadona” and “Coscia” pears by Stern et al. (2003) that TDZ treatment had no effect on fruit shape, seed number and yield in the following year. For the result that there was no difference in the seed number observed, the authors attributed this to low pollen utilization due to the lack of simultaneous flowering or the absence of pollinators. Nevertheless, our experiment does not have these problems and the research subjects are completely different, which may be the reason for the different results from the previous studies. Furthermore, both thousand-seed weight and seed volume weight reached their maximum values under the treatment with TDZ (200 μmol/L), and were higher than the control under other concentrations of TDZ treatment (Table 1). It was reported that TDZ treatment linearly increased the weight of bunches and berries, while reduced the total soluble solids content (Reynolds et al., 1992). Likewise, kiwifruit treated with different concentrations of TDZ were 50% or 60% heavier than untreated ones, the dry weight also increased substantially and to a similar increase extent at same treatment concentrations (Famiani et al., 1999). In our study, the yield of *B. napus* was higher than that of the control regardless of the application of any concentration of TDZ within the selected range of concentrations (Table 1). In terms of seed setting ratio of the whole plant, compared to the control, the seed setting ratio per plant was increased by 5.3 and 6.04% under low and high concentrations (10 and 400 μmol/L) of TDZ treatment, respectively (Supplementary Table 1). Consistent with previous studies, Pasa et al. (2017) found that the application of TDZ to “Hosui” and “Packham’s Triumph” pears significantly increased fruit setting, reduced fruit abscission and ultimately resulting in increased yield. Furthermore, in addition to “Hosui” and “Packham’s Triumph” pears, foliar sprays of TDZ were also observed in “Shinseiki” pears to significantly increase fruit setting (Hawerroth et al., 2011). Application of TDZ during flowering significantly increased fruit setting and fruit weight in apple trees “Gala” and “Fuji” over seven growing seasons. The 7-year average fruit setting ratio for TDZ 10 mg L^–1^ was 112.7% compared to 51.3% for the control (Petri et al., 2001). These results indicated that TDZ treatment increased yield traits such as the silique number per plant, thousand-seed weight, seed volume weight, the yield and seed setting ratio, thereby greatly improving the yield of *B. napus.*

### Thidiazuron treatments enlarger cell and increase the number of embryos of *Brassica napus*

Cell division and cell expansion control the size and shape of plant organs. In the present study, TDZ-treated rapeseed seeds were significantly larger than untreated seeds. The reason for this result could be that TDZ induced or prolonged the active phase of mitosis resulting in increased cell number, increased cell area or both (Celikel et al., 2021). The embryos of seeds in the siliques after 20 days of 200 μmol/L TDZ treatment were significantly larger than that of control (Figures 3A,E) and their average cotyledon area was 2.34 times larger than the control (Figure 4A). Similarly, embryos in mature seeds treated with 200 μmol/L TDZ were also significantly larger than the control (Figures 3C,G), and the average area of cotyledons after TDZ treatment was 1.81 times larger than that of control (Figure 4A). Both area and number of cotyledon epidermal cells increased after 200 μmol/L TDZ treatment (Figure 4B). Such a result demonstrated that exogenous application of TDZ induced an increase in cell size and number, which results in an increase in seed size of *B. napus*. Similar phenomena were observed in other plants. Shargal et al. (2006) suggested that the number of cells present at fruit setting, the number of subsequent cell divisions and cell expansion determined the fruit size of “Spadona” pears. The “Spadona” pear fruit treated with TDZ was significantly larger than the untreated fruit, and the cells between the parenchyma of the pulp were smaller but the number of parenchymal cells was significantly increased. On the other hand, according to the fluorescence-activated cell sorter (FACS) analysis, suggesting that TDZ affected pear fruit size by increasing cell number and prolonging the mitotically active period of pulp parenchyma cells (Shargal et al., 2006).

### Multiple seed development related pathways respond to thidiazuron treatments

**FIGURE 7:**
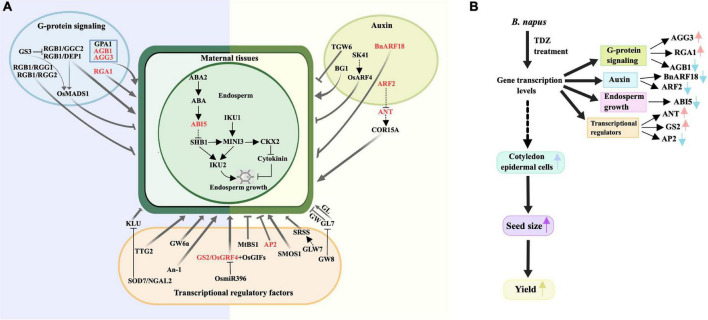
Diagram of signaling pathways associated with seed size **(A)** and a schematic diagram of a simulation mechanism explaining the regulation of seed size by TDZ **(B)**. **(A)** The image showed the genes related to the seed size in four different signaling pathways in *Arabidopsis*, rice and other species, where the red text represented the genes studied in this paper in *B. napus*, and the dashed lines indicated unclear genetic relationships (Li et al., 2019). ABA, abscisic acid; GL, grain length; GW, grain width. **(B)** TDZ treatment may initially affect changes in gene expression levels the yield of related to the seed size, resulting in larger cotyledon epidermal cells, increased seed size, and ultimately a substantial increase in *B. napus*.

**FIGURE 8:**
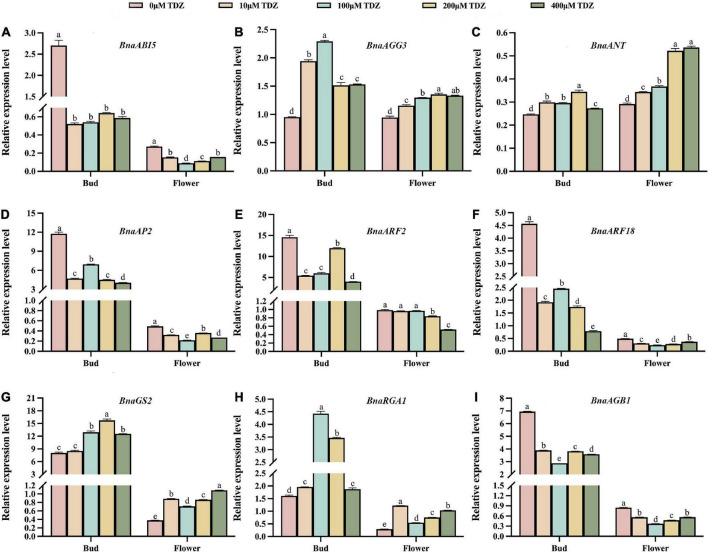
Comprehensive analysis of relative transcriptional levels of seed size-related genes in buds and flowers of *B. napus*. **(A–I)** lists the relative transcriptional levels of nine genes associated with seed size in buds and flowers. Among them, the pink, orange, blue, yellow and green columns represent the relative transcriptional levels of genes in buds and flowers treated with 0 μmol/L, 10 μmol/L, 100 μmol/L, 200 μmol/L and 400 μmol/L TDZ, respectively. Three biological replicates and three technical replicates were carried out using buds from 3 to 5 days before flowering and 10-day-old blooming flowers. Mean ± Standard error (*n* = 3). Different letters suggest significant differences (Duncan’s multiple range test, *p* ≤ 0.05).

A previous review showed that seed size is controlled by a combination of several different signaling pathways that regulate maternal tissues growth (Li et al., 2019). Figure 7A showed a partial signaling pathways diagram related to seed size involved in this study. The latest study found that KIX8/9 and PPD1/2 led to larger seeds by increasing cell proliferation and cell elongation, suggesting that the KIX-PPD-MYC-GIF1 pathway has a role in controlling seed size (Liu et al., 2020). Our real-time qPCR data revealed that these nine genes directly related to endosperm growth, G-protein signaling, auxin metabolic pathways and transcription factors controlling seed size were differentially regulated by TDZ in buds and flowers of *B. napus* at early developmental stages. Figure 7B is a schematic diagram of the simulation mechanism explaining how TDZ regulates the seed size of *B. napus*. Specifically, three genes (*AGB1*, *AGG3*, and *RGA1*) were identified in G-protein signaling, two genes (*BnARF18* and *ARF2*) in auxin metabolic pathway, three genes (*ANT*, *AP2*, and *GS2*) in transcriptional regulatory factors and an endosperm growth-related gene (*ABI5*). In order to more intuitively reflect the difference in the overall expression level of each gene between different tissues, we summed up the expression levels of genes located on different chromosomes. Figure 8 showed the trend of the overall expression of each gene in buds and flowers after different concentrations of TDZ treatment. It can be seen from the figure that the change trend of the overall expression level of a single gene in different tissues is the same as the change trend of the expression level of the gene located on different chromosomes. Meanwhile, the expression patterns of these genes were similar in the buds and flowers. Exogenous application with TDZ in bud down-regulated the expression of auxin metabolic pathway genes, such as *ARF2* and *BnARF18*. Schruff et al. (2006) reported that *ARF2* was a suppressor of cell division, tissue development and organ growth, and it determined seed size and weight by regulating integument growth and development. Subsequently, *ARF2* was shown to negatively regulate the expression of the homologous structural domain gene *HB33*, thereby controlling ABA-mediated primary root growth and seed germination (Wang et al., 2011). A recent study demonstrated that *ARF2* could negatively regulate the expression of *ANT* after binding to the *ANT* promoter region, which in turn regulated seed weight and drought resistance by promoting cell proliferation (Meng et al., 2015). In addition, *ARF18* was showed as a transcriptional repressor, which can activate downstream auxin genes to increase cell size, thereby regulating SL and SW (Liu J. et al., 2015). Our results also suggested that the expression levels of several genes involved in endosperm growth and G-protein signaling were down-regulated in response to TDZ treatment, such as *ABI5*, *AP2*, and *AGB1* (Figure 7). *ABI5* has the effect of attenuating or terminating ABA signaling during seed germination, hence *ABI5* can regulate seed dormancy, germination and seedling growth (Lopez-Molina et al., 2001). In *Arabidopsis*, ABA downstream signaling component *ABI5* have been proposed to decrease the expression of *SHB1* by combining with the *SHB1* promoter region during early seed development, which caused the seed size increased in abi5 (Cheng et al., 2014). The Gβ subunits in the heterotrimeric G protein complex play important roles in seed germination, seed size, stress adaptation, and stomatal opening and closing (Ullah et al., 2002; Xu et al., 2015). *AGB1* has been shown previously to function as a negative regulator in ABA responses (Pandey et al., 2006). The plasticity of fruit number, seed number per fruit and total seed yield were reduced in the agb1 mutant compared to Col (Nilson and Assmann, 2010). Interestingly, recent researches indicated that the transcription factor AP2/ERF-domain family gene *SIDREB3* also negatively regulated ABA responses in tomato and its over-expression reduced final fruit size (Gupta et al., 2022). In *Arabidopsis*, *AP2* was shown to be a negative regulator of seed size and weight. When *AP2* gene expression decreased, *ap2* mutants produced abnormally shaped large seeds in which not only the seed size and weight increased, but also the number and size of embryo cells increased simultaneously due to endosperm cellularization and outward growth of the central endosperm vacuole (Jofuku et al., 2005; Ohto et al., 2005, 2009). Thus, *AP2* has a negative regulatory effect on seed size and weight. Additionally, *AP2* genes have been found to have similar effects in other plant species, such as Larix Li et al. (2013) and *Aechmea fasciata* (Lei et al., 2019). Two G protein signaling-related genes and two transcriptional regulatory factors, such as *AGG3*, *RGA1*, *ANT*, *GS2* were up-regulated by TDZ treatment. Prior research in rice highlighted the importance of *RGA1* in dwarfism and set small seed, and this study identified *RGA1* as a positive regulator of cellular proliferation and dwarfism in d1 was caused by a decrease in the number of cells (Izawa et al., 2010). Consistent with previous studies, which demonstrated a positive regulatory role for *AGG3* in the control of seed and organ size in *Arabidopsis*, *AGG3* overexpression resulted in increased seed size, fruit length, and number of seeds per fruit. Instead, *AGG3* loss-of-function mutants had smaller seeds and reduced number of seeds per fruit (Chakravorty et al., 2011; Li et al., 2012; Roy Choudhury et al., 2014). The *ANT* gene is a key transcription factor for ovule development and organ growth in *Arabidopsis*. It has been proved that after overexpression of the *ANT* gene driven by the 35s promoter, ant mutants exhibit a larger seed phenotype due to cell expansion (Mizukami and Fischer, 2000). Recently, several researches have reported that when *GS2*/*OsGRF4* directly interacted with the transcriptional co-activator *OsGIF1*, the elevated expression levels of both resulted in increased cell size and number, which positively regulated grain size and grain weight, and significantly improved yield (Che et al., 2016; Duan et al., 2016; Li et al., 2016).

## Conclusion

Thidiazuron treatment could increase the seed diameter and silique length of *B. napus* to varying degrees and greatly improve rapeseed yield. TDZ increased seed size by promoting cell elongation, cell division and regulating gene expression in several signaling pathways associated with maternal tissue growth. This study preliminarily explored the mechanism of TDZ treatment on the seed size of *B. napus* and provided an important reference for improving rapeseed yield.

## Data availability statement

The original contributions presented in this study are included in the article/Supplementary material, further inquiries can be directed to the corresponding authors.

## Author contributions

BX, JD, and XZ: designed the research. LZ: conducted the research and analyzed the data. JX: supplied the materials. LZ, LX, BX, JX, JD, and XZ: wrote and edited the manuscript. All authors scrutinized and corrected the manuscript.

## Abbreviations

*B. napus*: *Brassica napus L.*
TDZ: thidiazuron
GA_3_: gibberellic acid 3
DIC: differential inference lens
EDTA: ethylene diamine tetraacetic acid
DMSO: dimethyl sulfoxide
RT-qPCR: real time quantitative PCR
SS: seed number per silique
SL: silique length
SW: seed weight
BnPIR: *Brassica napus* Pan-genome Information Resource
BLAST: Basic Local Alignment Search Tool.
