# Supplementary material for: Study on exogenous application of thidiazuron on seed size of Brassica napus L
Source: Front Plant Sci. 2022 Sep 6;13:998698. doi: 10.3389/fpls.2022.998698 (PMC9486165; doi:10.3389/fpls.2022.998698)
Supplement: Supplementary file 1 [file Data_Sheet_1.docx]

**Table S1.** Effects of TDZ Treatment on seed morphology and quantity in *B. napus*.

| location | Treatment  (µmol/L) | Seed diameter  (mm) | Seed number  per silique | Empty shells number | Seed setting ratio (%) |
| --- | --- | --- | --- | --- | --- |
| Top | Control | 1.93±0.03b | 18.48±0.97b | 9.55±0.73 | 65.54±2.73 |
|  | TDZ 10 | 1.97±0.03ab | 19.67±0.92ab | 9.37±0.63 | 67.37±2.18 |
|  | TDZ 100 | 2.04±0.03a | 19.20±1.06b | 10.30±0.66 | 64.20±2.47 |
|  | TDZ 200 | 2.05±0.04a | 20.10±1.15ab | 11.20±0.80 | 63.65±2.65 |
|  | TDZ 400 | 2.03±0.03a | 22.30±0.81a | 10.50±0.61 | 68.48±1.79 |
| Middle | Control | 1.92±0.03b | 19.79±0.87b | 10.72±0.93a | 65.39±2.65 |
|  | TDZ 10 | 1.95±0.03b | 20.10±0.93b | 8.33±0.73b | 70.67±2.51 |
|  | TDZ 100 | 1.96±0.03ab | 19.82±0.84b | 9.82±0.92ab | 67.39±2.85 |
|  | TDZ 200 | 2.04±0.03a | 22.13±0.76ab | 10.13±0.47ab | 68.34±1.54 |
|  | TDZ 400 | 2.01±0.02ab | 23.23±0.57a | 9.27±0.62ab | 72.11±1.39 |
| Bottom | Control | 1.99±0.03b | 18.87±0.62b | 12.23±0.69a | 60.96±1.84b |
|  | TDZ 10 | 2.02±0.03ab | 19.53±0.78ab | 9.07±0.64b | 68.35±2.07a |
|  | TDZ 100 | 2.10±0.04a | 18.77±0.72b | 9.93±0.78b | 65.76±2.52ab |
|  | TDZ 200 | 2.05±0.04ab | 20.97±0.64a | 10.70±0.60ab | 66.15±1.33ab |
|  | TDZ 400 | 2.01±0.02ab | 20.60±0.63ab | 9.80±0.60b | 68.02±1.65a |
| Various portion | Control | 1.96±0.02b | 19.28±0.50c | 11.09±0.45a | 63.50±1.35b |
|  | TDZ 10 | 1.97±0.02b | 19.77±0.50bc | 8.92±0.38c | 68.80±1.30a |
|  | TDZ 100 | 2.03±0.02a | 19.01±0.49c | 9.77±0.46bc | 66.21±1.54ab |
|  | TDZ 200 | 2.05±0.02a | 20.97±0.52ab | 10.68±0.37ab | 66.04±1.12ab |
|  | TDZ 400 | 2.01±0.01ab | 21.76±0.40a | 9.86±0.35bc | 69.54±0.94a |

Note: Seed diameters, seed number per silique (SS), empty shells number and seed setting ratio located in various portion of *B. napus* treated with different concentrations of TDZ were measured at the time of full ripeness of the silique. The data listed in the table represents the mean ± SE. Means with the different letter differed significantly according to the Duncan's test (p ≤ 0.05). Each data comes from the average of at least five plants.

**Table S2.** Primers used for quantitative real time RT-PCR analysis.

| S.No. | Gene | Locus | Forward Primer | Reverse Primer |
| --- | --- | --- | --- | --- |
| 1 | *BnaA05.ABI5* | *BnaA05G0087100ZS* | TAAGCCCTGTGTCGTCTGATGG | TCATCCTCCTCTGCCTTCTCTC |
| 2 | *BnaC04.ABI5* | *BnaC04G0102500ZS* | AGCAGCCGAGTCTTCCACGA | AAGTGTCATCTCCCCGAACG |
| 3 | *BnaC09.AGG3* | *BnaC09G0459200ZS* | AAGTTGCTGCTGCGTTGGGTCC | GTCGAGAGCAACTGCGGAAAC |
| 4 | *BnaA10.AGG3* | *BnaA10G0172300ZS* | AACCAACAAGATTCCCTCCACT | ATGAGAGGAGGTTGGAAGCC |
| 5 | *BnaA01.ANT* | *BnaA01G0012600ZS* | CGGTAGAGTCGCTGGAAACAAAG | TTCCGACCACATTTGCTCCG |
| 6 | *BnaA08.ANT* | *BnaA08G0188100ZS* | CAGAACCAAAACCACCAAGGC | AGTCTGCTGCTTCTGTCCAAC |
| 7 | *BnaA01.AP2* | *BnaA01G0016800ZS* | AGACGCTGTGACTAACTTTGAT | ATGAGAGAAGGTTGGAAGCC |
| 8 | *BnaC01.AP2* | *BnaC01G0019400ZS* | AAGTGTGGTCGTTGGGAAGC | GTTGGTTCATCTTGAGCCGC |
| 9 | *BnaA03.AP2* | *BnaA03G0561000ZS* | GCATAAGTGTGGTCGCTGGG | CGGCATTCAGTTCTTCGTCG |
| 10 | *BnaC03.ARF2* | *BnaC03G0559800ZS* | GGCTCCTAAGAAAGATTGGCTGA | GTTCTTCCTCGTTCCTACAGCATA |
| 11 | *BnaA06.ARF2* | *BnaA06G0273300ZS* | GACCGTTTATCATCATCACCTTC | CGTTGTTTACCAGAGGAATGCC |
| 12 | *BnaA04.ARF18* | *BnaA04G0008800ZS* | GAGCCAACAACATTTCAGAGACC | CCATACAAGAAAGAAGGCGGAGC |
| 13 | *BnaC04.ARF18* | *BnaC04G0265800ZS* | CAATGCCTACTTCGGTTATCTCG | TGACACCTACTATGAACTGGCTT |
| 14 | *BnaC08.ARF18* | *BnaC08G0407500ZS* | GATGCCTGCTTCGGTTATTTCG | CTTGTCTGGAGGTGTTGGAGC |
| 15 | *BnaA08.GS2* | *BnaA08G0077600ZS* | GAGAGAGGAAGGAGGATTTGATG | TGAGCATCCACGGTTAGCC |
| 16 | *BnaC08.GS2* | *BnaC08G0109700ZS* | GAGAGGAAGGAGGATTTGATGTG | GAGCATCCACGGTTAGCCA |
| 17 | *BnaC04.GS2* | *BnaC04G0369400ZS* | GACGGAGGATTTGAGGTGATTA | ATGTTAGACGCTGGACGCCGAT |
| 18 | *BnaC09.RGA1* | *BnaC09G0254100ZS* | CGCTGCATTACTATTCGACACTG | AACACCCATTATTCTCCTCCACA |
| 19 | *BnaC07.RGA1* | *BnaC07G0269400ZS* | AGCGAAACCGAATCTGTGGCG | CGGACCTTCGCAAGCCAC |
| 20 | *BnaA06.RGA1* | *BnaA06G0409200ZS* | CTACGAGACTTGCCCTTACCT | TCGGTTTCACTCGGTCTAAGC |
| 21 | *BnaC01.AGB1* | *BnaC01G0042900ZS* | ATCAACTCCAGGTGTATCAGCC | CTCCTGTGCCCTCCAAACG |
| 22 | *BnaC07.AGB1* | *BnaC07G0521400ZS* | GGCTTCTCTTCGCTGGCTACG | CACTCCCATCTGCTGACATCC |
| 23 | *BnaA08.AGB1* | *BnaA08G0135000ZS* | GCAAGCAGAGCAGTGGGGACG | GCGTAGCCAGCGAAAAGAAGCC |
| 24 | *BnaA01.AGB1* | *BnaA01G0037500ZS* | GGCACGAGGGAGATGTCAATAC | CCCAAATCCAATACAATCTCTCC |
| 25 | *BnaC03.AGB1* | *BnaC03G0746200ZS* | ATTTCTGTCTTTGGCGGCGAG | TCTCCCTTGTGCCCATGAAACG |
| 26 | *Bnactin* | *NP_001302489* | TTGGAACGATACAGAGAAGATTAGCA | TTGGACCATTTCTCGATTTGTG |
